# Structure Engineering in Biomass-Derived Carbon Materials for Electrochemical Energy Storage

**DOI:** 10.34133/2020/8685436

**Published:** 2020-04-29

**Authors:** Ruizi Li, Yanping Zhou, Wenbin Li, Jixin Zhu, Wei Huang

**Affiliations:** ^1^Frontiers Science Center for Flexible Electronics (FSCFE), Shaanxi Institute of Flexible Electronics (SIFE) & Shaanxi Institute of Biomedical Materials and Engineering (SIBME), Northwestern Polytechnical University (NPU), 127 West Youyi Road, Xi'an 710072, China; ^2^College of Electronics and Information Engineering, Sichuan University, No. 24 South Section 1, Yihuan Road, Chengdu 610064, China; ^3^Institute of Advanced Electrochemical Energy & School of Materials Science and Engineering, Xi'an University of Technology, Xi'an, Shaanxi 710048, China; ^4^Key Laboratory of Flexible Electronics (KLOFE) & Institute of Advanced Materials (IAM), Jiangsu National Synergetic Innovation Center for Advanced Materials (SICAM), Nanjing Tech University (NanjingTech), 30 South Puzhu Road, Nanjing 211816, China

## Abstract

Biomass-derived carbon materials (B-d-CMs) are considered as a group of very promising electrode materials for electrochemical energy storage (EES) by virtue of their naturally diverse and intricate microarchitectures, extensive and low-cost source, environmental friendliness, and feasibility to be produced in a large scale. However, the practical application of raw B-d-CMs in EES is limited by their relatively rare storage sites and low diffusion kinetics. In recent years, various strategies from structural design to material composite manipulation have been explored to overcome these problems. In this review, a controllable design of B-d-CM structures boosting their storage sites and diffusion kinetics for EES devices including SIBs, Li-S batteries, and supercapacitors is systematically summarized from the aspects of effects of pseudographic structure, hierarchical pore structure, surface functional groups, and heteroatom doping of B-d-CMs, as well as the composite structure of B-d-CMs, aiming to provide guidance for further rational design of the B-d-CMs for high-performance EES devices. Besides, the contemporary challenges and perspectives on B-d-CMs and their composites are also proposed for further practical application of B-d-CMs for EES devices.

## 1. Introduction

With the explosive growth of global economy and population, the energy consumption worldwide has attracted more and more attention [1]. The extensive use of fossil fuels has not only led to its depletion but also brought about severe environmental problems such as global warming, forest damage, air pollution, and acid rain [2, 3]. As such, the exploration of green and sustainable energy including hydropower, tidal energy, and solar energy is stringent. However, these renewable energy sources suffer from intermittence, where highly efficient energy storage technique is very desirable to achieve a continuous and more reliable supply in practical applications [4].

Among various energy storage systems, electrochemical energy storage (EES) devices, such as sodium-ion batteries (SIBs) [5], lithium-sulfur (Li-S) batteries [6], and supercapacitors [7], have shown large potential and attracted extensive research interests. Specifically, SIBs are viewed as an appealing counterpart for lithium-ion batteries due to the abundance, democratic distribution, and low cost of sodium resource, as well as the similarity of sodium to lithium in physiochemical properties [8]. Li-S batteries have high energy density (2600 Wh kg^−1^) and theoretical capacity (1675 mAh g^−1^) [9], while supercapacitors are well known for their excellent reversibility and high power density [10]. The performances of EES devices including cost-effectiveness, gravimetric/volumetric energy density, cycling stability, and rate response all rely severely on features of electrode materials.

Up to now, numerous electrode materials have been explored for EES, primarily including carbonaceous materials [11], Si/Ge/Sn [12], transition metal oxides/sulfides [13, 14], and MXene [15]. Among them, carbonaceous materials become a group of very promising electrode materials by virtue of their high electric conductivity, large specific surface area, outstanding structural and chemical stability, controllable pore size distribution, and good mechanical strength [16, 17]. However, the wide practical usages of high-quality carbonaceous materials (e.g., graphene, fullerene, and carbon nanotube) are greatly hindered by high requirement of equipment and low production yield [18]. Sometimes, the preparation processes inevitably utilize toxic and dangerous reagents as well as pollutants [19]. Carbon materials derived from renewable biomass are highly desirable, because of their naturally diverse and intricate microarchitectures, extensive and low-cost source, environmental friendliness, and feasibility to be produced in a large scale [20–22]. More importantly, their large interlayer distances, disordered structures, and abundant active functional groups can provide more potential charge storage sites [23–25]. In addition, various facile and green methods have been proposed to convert biomass into value-added carbon materials without using any expensive chemical reagents and complex installations, including one-step pyrolysis, hydrothermal carbonization, physical and chemical activations, molten salt carbonization, and template method [26–31]. As such, biomass-derived carbon materials (B-d-CMs) have been regarded as promising candidates for EES devices [32–54].

Energy storage mechanisms of B-d-CMs are highly dependent on their EES devices. Specifically, B-d-CMs usually show two kinds of Na^+^ storage mechanisms: (1) the insertion/extraction in the interlayer of the graphitic crystallites at low potentials (below 0.1 V), relating to the plateau region of charge/discharge curves, and (2) the electroadsorption/desorption at active functional groups, structural defect sites, and pore surfaces at high potentials (above 0.1 V), corresponding to the sloping region of charge/discharge curves [55]. Firstly, the hard carbon feature of B-d-CMs presents larger interlayer spacing than graphite, which is conducive to the insertion/extraction of Na^+^. Moreover, the abundant pore structure and large specific surface area of B-d-CMs are in favor of Na^+^ storage by electroadsorption/desorption. However, the lower electronic conductivity caused by the less graphitized region and unstable surface functional groups of raw B-d-CMs, coupled with larger radius of Na^+^ (0.102 nm) than Li^+^ (0.076 nm), gives rise to fewer storage sites and slower diffusion kinetics for Na^+^ insertion/desertion reaction and adsorption/desorption process. This largely limits the further development of raw B-d-CMs in SIBs [8]. In the lithium-sulfur battery system, the mainly electrochemical reaction is S + 2Li^+^ + 2e^−^⟷Li_2_S. The insulating nature of S and the shuttling of intermediate polysulfides lead to poor charge/electron transfer, loss of active material, passivation of Li anode, increasing electrolyte viscosity, etc. It will consequently induce fast capacity decay and poor cycling life and rate performance [56]. B-d-CMs could modulate the electronic property of the S-containing cathode, enhance the affinity for polysulfides to the cathode through additional chemisorption, and thus improve their performance. However, the ability of raw B-d-CMs to enhance the Li-S battery performance is unsatisfactory due to their low content of heteroatoms and functional groups, as well as poor storage sites and diffusion kinetics. Supercapacitors are divided into two families: pseudocapacitor and electric double-layer capacitor (EDLC). In EDLC, B-d-CMs act as active material by charging of the double-layer capacitance via the reversible ion adsorption/desorption on the carbon surface [57]. Although the raw B-d-CMs have fast charge-storage capacity in EDLC, the less charge-storage sites result in low capacity and largely restrict their practical application [58]. Consequently, the practical application of raw B-d-CMs in EES devices is mainly hindered by their limited number of efficient storage sites and diffusion kinetics as electrodes in SIBs, Li-S batteries, and supercapacitors.

**Figure 1:**
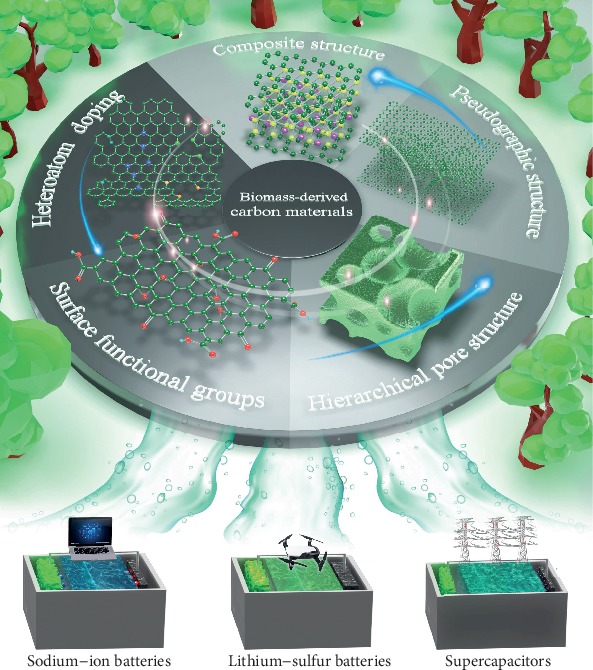
Schematic illustration of the structure optimization strategies of B-d-CMs and their application in various EES devices.

In recent years, great efforts have been devoted to enhancing the electrochemical energy storage performance of B-d-CMs. Based on them, the structural diversities (i.e., 1D, 2D, and 3D), synthetic methods, and specific application of B-d-CMs in one type of EES device have been summarized in some previous reviews [24, 25, 59–71]. The controllable storage sites and diffusion kinetics to boost B-d-CMs for EES devices have not been well reviewed. So, it is very necessary and timely to comprehensively review the structure design of B-d-CMs affecting EES performances when promoting the storage sites and diffusion kinetics in energy storage devices. In this review, a controllable design of B-d-CM structures boosting its storage sites and diffusion kinetics for EES devices, including SIBs, Li-S batteries, and supercapacitors, is reviewed from the aspects of effects of pseudographic structure [28, 72–74], hierarchical pore structure [75–80], surface functional groups [81–84], and heteroatom doping [85–95], as well as the composite structure of B-d-CMs [96–99] as shown in Figure 1, aiming to promote controllable design of effective B-d-CMs for EES devices. Besides, the contemporary challenges and perspectives in B-d-CMs and their composites are also proposed for further rational design of B-d-CMs for EES devices.

## 2. Controllable Design of B-d-CM Structure Boosting Its Storage Sites and Diffusion Kinetics

### 2.1. Pseudographitic Structure

The pseudographitic structure of B-d-CMs usually refers to the disordered turbostratic nanodomains with graphitic layers and a few uniformly and randomly arranged defects. It can largely affect the storage sites and diffusion kinetics of B-d-CMs [74]. The disordered turbostratic nanodomains can not only provide abundant ion-storage sites between the graphitic layers but also afford the pathway for electron transport and ion diffusion, contributing to the storage of more ions and the reduction of the energy barrier for ion insertion/extraction. Thus, enlarging the graphitic interlayer spacing and increasing the order and number of turbostratic nanodomains can collectively promote ion-storage sites and diffusion kinetics and enhance the specific capacity, cycling, and rate performance [100, 101].

**Figure 2:**
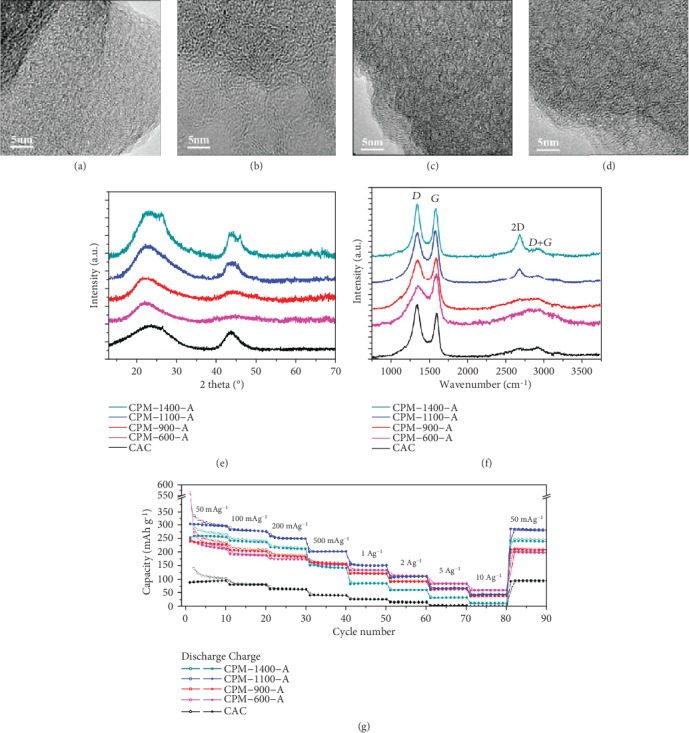
(a–d) HRTEM images. (e) XRD patterns. (f) Raman spectra of peat moss-derived carbon materials. (g) Rate performance of peat moss-derived carbon as anode in SIBs [72] (copyright 2013, American Chemical Society).

Ding and coworkers [72] synthesized carbon nanosheet frameworks from peat moss with a highly ordered pseudographic structure (Figures 2(a)–2(f)) via a pyrolysis process followed an air activation step and investigated their Na^+^ storage properties (Figure 2(g)). Benefitting from the highly ordered pseudographite arrays and substantially large interlayer spacing of 0.388 nm, the carbon nanosheet frameworks exhibited significantly excellent Na^+^ storage kinetics and intercalation capacity in SIBs. Lotfabad et al. [28] fabricated the banana peel derived hard carbon with ordered pseudographitic structure though a pyrolysis procedure followed by air activation. Owing to the pseudographitic structure with a larger interlayer spacing of 0.392 nm that could facilitate more Na^+^ insertion/extraction, the as-obtained banana peel-derived hard carbon showed excellent electrochemical performance, delivering a stable cycling capacity of 336 mAh g^−1^ after 300 cycles at 50 mA g^−1^. Wang et al. [102] prepared graphitic carbon nanosheets from cornstalk biomass through an in situ self-generating template method. Due to the ordered pseudographitic structure, the sample showed excellent conductivity in supercapacitors, enabling superior capacitance. Sun et al.'s group [103] reported the facilely synthetic process of hard carbon from shaddock peels for SIBs via one-step pyrolysis processes. Because the synergistic effect of its honeycomb-like morphology and pseudographitic structure, the pyrolytic sample displayed a high specific capacity of 430.5 mAh g^−1^ at 30 mA g^−1^.

**Figure 3:**
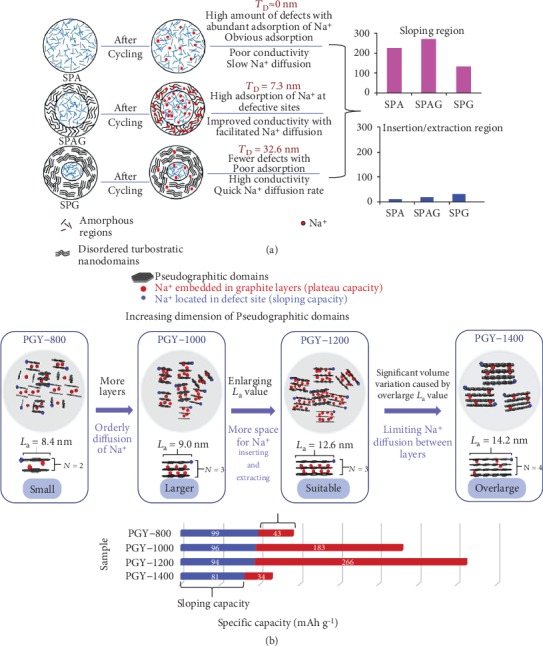
(a) Graphic illustration of microstructure evolution for hard carbon from the shaddock peel as electrodes before and after cycling [104] (copyright 2018, Wiley). (b) Graphic illustration of Na^+^ insertion into a pseudographitic domain [105] (copyright 2017, Elsevier).

Notably, the highly ordered pseudographitic structure (more nanodomain) is usually obtained at high temperature, which negatively decreases the interlayer spacing, specific surface area, and amorphous carbon region, thus providing poor storage sites [60]. Li et al.'s group [104] achieved a balance between the pseudographitic structure (disordered turbostratic nanodomains) and amorphous carbon structure by controlling the reaction temperature to obtain the pseudographitic structure with different thicknesses.  Figure 3(a) exhibited the hard carbon from shaddock peel developed at 500°C, 600°C, and 700°C (designated as SPA, SPAG, and SPG, respectively), with the disordered turbostratic nanodomains (*T*_D_) thickness of 0, 7.3, and 32.6 nm, respectively. SPA was almost entirely amorphous carbon structure with numerous defect sites, leading to a poor conductivity and slow Na^+^ diffusion rate. SPG with a high *T*_D_ value displayed a small amount of amorphous structure, resulting in fewer electroactive sites, less Na^+^ adsorption, and lower capacity than the other two samples. SPAG presented a suitable *T*_D_ value to keep the high diffusivity of Na^+^ and electron transmission, consequently balancing the charge conductivity, Na^+^ diffusion kinetics, and the number of adsorption sites and then showing a high electrochemical performance. Besides, Wang et al.'s group [105] also optimized the pseudographitic domain dimension from dandelion, as shown in Figure 3(b). The as-obtained sample synthesized at 1000°C had a bigger length of average width (*L*_a_), number of graphite layers (*N*), and thickness (*L*_c_) than that obtained at 800°C, thus exhibiting a higher degree of graphitization and higher diffusion kinetics. When the reaction temperature rose to 1200°C, *L*_a_ continued to grow but *L*_c_ and *N* values no longer changed. The enlarged width of the pseudographitic structure provided enhanced diffusion kinetics and more active sites for Na^+^ storage in insertion process. Moreover, the values of *N* and *L*_a_ were significantly increased with the rising of temperature to 1400°C, which resulted in a low capacity because of the deceased interlayer spacing. The sample prepared at 1200°C balanced the interlayer distance and pseudographitic domain dimension to increase the Na^+^ storage sites, hence delivering a high capacity of 364.3 mAh g^−1^ after 10 cycles at 50 mA g^−1^.

### 2.2. Hierarchical Pore Structure

Hierarchical pore structure, containing micropores (below 2 nm), mesopores (2~50 nm), and macropores (above 50 nm), is conducive to improving the diffusion kinetics and increasing the storage sites of electrode materials in practical application of EES devices [106]. Specifically, macropores are equivalent to an ion-buffering reservoir that can minimize the distance for ion diffusion to the interior surfaces of B-d-CMs. Mesopores and micropores could supply a large surface area for electrolyte-electrode material interaction and provide a low-resistant transfer pathway for electrolyte ions [107, 108]. Thus, constructing a well-defined hierarchically porous structure can not only contribute to a high specific surface area with abundant storage sites leading to high energy density and great capacitance but also shorten the distance for ion diffusion with enhanced diffusion kinetics enabling improved rate capability and power density [109]. Although there are intrinsic pores in raw B-d-CMs, the development of a well-defined pore structure for the design of high-performance EES devices with numerous storage sites and high diffusion kinetics is urgently needed [26].

Recently, various pore-creating techniques have been developed, which could control and design a well-defined hierarchically porous structure from various biomass sources [75–80]. Generally, the synthetic methods of the hierarchically porous structure can be classified into two categories: physical activation and chemical activation. In physical activation, CO_2_, H_2_O steam, ozone, or air are usually used to activate B-d-CMs at high temperature usually above 700°C [110]. In chemical activation, B-d-CMs are premixed with a chemical agent (KOH, KHCO_3_, K_2_CO_3_, NaOH, NaHCO_3_, FeCl_3_, ZnCl_2_, or H_3_PO_4_ [111]) and subsequently thermally treated usually in the range of 500~1000°C. Compared with physical activation, chemical activation is more widely applied due to the lager specific surface area, lower reaction temperature, higher yields, and lower cost of the products. Using biomass as a precursor, the electrode materials with a well-defined hierarchical porous structure formed by chemical activation can significantly enhance the storage sites and diffusion kinetics in SIBs, Li-S batteries, and supercapacitors.

**Figure 4:**
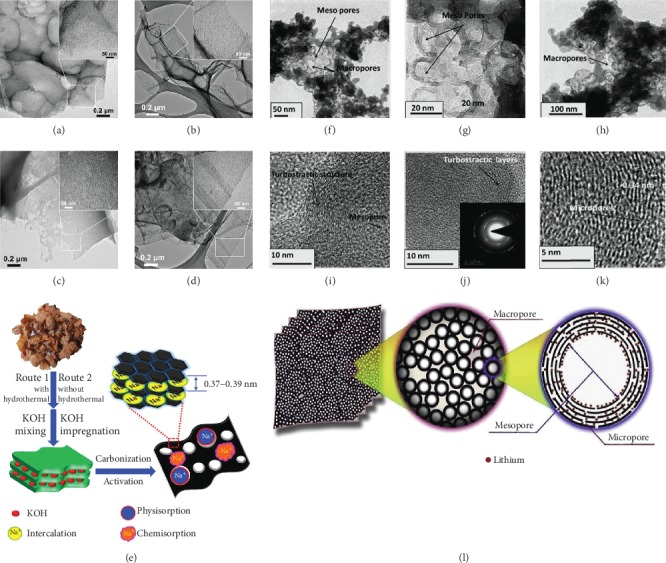
(a–d) TEM micrographs and (e) graphic illustration of the formation of hierarchical porous carbon from peanut skin. (f–h) TEM and (i–k) HRTEM images of prawn shell-derived carbon materials [29] (copyright 2015, Elsevier). (l) Mechanism of Li/Na insertion in prawn shell-derived carbon materials as electrodes [44] (copyright 2016, Elsevier).

In SIBs, B-d-CMs with hierarchical pore structures have been well designed to enhance the storage sites and diffusion kinetics by supplying numerous channels in the carbon to shorten the Na^+^ diffusion path in insertion/extraction process, providing efficient active pores for Na^+^ accommodation and promoting charge across the electrode/electrolyte interface. For instance, the carbon material derived from peanut skin as shown in Figures 4(a)–4(e) was synthesized [29] through pyrolysis followed by chemical activation (KOH). The as-prepared material showed sheet-like morphology with interconnected hierarchical pore structures and a large surface area (2500 m^2^ g^−1^). Tested against sodium, it exhibited good rate capability and cycling stability. Prawn shell was selected as another ideal biomass candidate [44]. The prawn shell-derived carbon possessed a distinct porous structure with macro-, meso-, and micropores, which could generate quick Na^+^ storage and diffusion in SIBs, as shown in Figures 4(f)–4(l).

As for supercapacitors, the hierarchically porous architecture could improve the pseudocapacitors, because the hierarchical pore structure offers quick ion/e^−^ transmission and alleviates structural degradation induced by volume expansion [114]. For example, hierarchically porous carbon nanosheet [115] had been obtained through synchronous activation (by FeCl_3_ and ZnCl_2_) and graphitization of natural silk. It exhibited a high volume of hierarchical pores (2.28 cm^3^ g^−1^) and high specific surface area (2494 m^2^ g^−1^) and delivered a high capacitance and excellent cycling stability (9% loss after 10000 cycles). Chen and coworkers [116] prepared an activated carbon from cotton stalk with H_3_PO_4_ as the chemical activation reagent. The as-obtained carbon with a surface area of 1481 cm^2^ g^−1^ and pore volume of 0.0377 cm^3^ g^−1^ displayed a capacitance value as high as 114 F g^−1^ at 0.5 A g^−1^ owing to its unique structure features.

**Figure 5:**
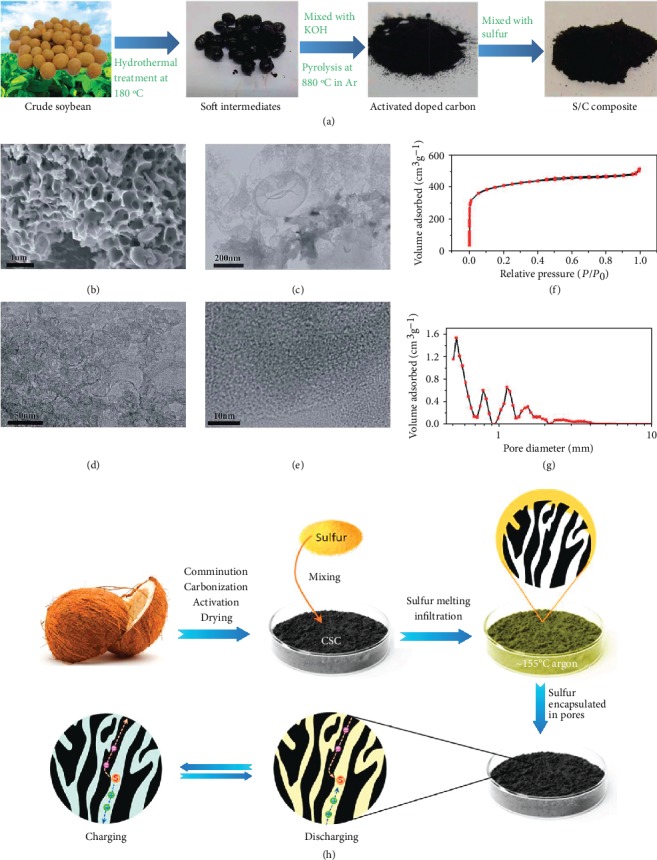
(a) Schematic of the preparation procedure, (b) SEM image, (c–e) TEM images, (f) nitrogen adsorption/desorption isotherm, and (g) pore size distribution of the soybean-derived porous carbon [112] (copyright 2016, Royal Society of Chemistry). (h) Schematic diagram for the preparation of S/coconut shell carbon [113] (copyright 2017, American Chemical Society).

Trapping the polysulfides, improving the electrical conductivity, increasing efficient reaction sites, and elevating diffusion kinetics of the sulfur electrode in the conductive matrix are vitally important to enhance the electrochemical performance for Li-S batteries. By the help of the hierarchically porous structure, large surface area, and low cost, B-d-CMs are appropriate for solving this problem. The soluble polysulfide intermediates could be effectively trapped in the porous carbon, which could guarantee the sufficient storage sites for electrochemical reaction and effectively improve the quality of electrolyte for Li^+^ diffusion. Meanwhile, the carbon materials can supply a useful electron conducting network and elevate the utilization efficiency of sulfur in the electrode. Soybean-derived hierarchically porous carbon [112] as shown in Figures 5(a)–5(g) had been prepared for Li-S batteries. The obtained carbon displayed large capacity, high Coulombic and energy efficiencies, and high cycling stability, which could be associated with its high specific surface area (1500 m^2^ g^−1^) and hierarchical microporous/mesoporous structure. Chen et al.'s work [113] further revealed that hierarchical pore structures were highly desirable for Li-S batteries in Figure 5(h). Micro-/mesoporous coconut shell carbon was favorable to repress the “shuttle effects.” The sulfur-infiltrated materials showed a larger specific capacity of 1599 mAh g^−1^ at 0.5 C.

In addition, the unique 3D-interconnected hierarchical porous structure is also very significant, which could supply 3D paths for the electrolyte diffusion and structure stability, highly improving the storage sites and diffusion kinetics [117]. Zhang et al. [36] synthesized a 3D connected hierarchically porous carbon foam via the pyrolysis and KOH activation process of pomelo peel. The impregnation of sulfur into the micro-/mesopores was considered to be a good way for efficient sulfur utilization, providing enough storage sites. When used in Li-S batteries, it delivered an initial discharge capacity of 1258 mAh g^−1^ at 0.2 C.  Table 1 presents the comparison of the pseudographitic structure and hierarchically porous structure of B-d-CMs and their electrochemical performance in various EES devices.

### 2.3. Surface Functional Groups

Except for the controllable design of the pseudographic structure and hierarchically porous structure, surface functional groups (surface chemistry and energy) of B-d-CMs are also essential to the improvements of efficient storage sites and diffusion kinetics in EES devices [81–84]. Surface functional groups could provide numerous electrochemical active sites, playing an important role in the surface-adsorption processes and reversible surface redox reactions, which compared with the ion-intercalation reaction could facilitate more storage sites and faster ion diffusion, as well as smaller electrode structure damage. This will be greatly beneficial for high reversible capacity and excellent rate performances [120–123]. Raw B-d-CMs usually contain certain amounts of oxygen, nitrogen, or sulfur-related functional groups. However, not all functional groups are favorable for electrochemical performance. For example, while C=O could generate surface reactions with Na^+^ by C = O + Na^+^ + e^−^⟷C − O − Na, leading to the increase of reversible capacity, while C-O and -COOH will result in a poor Coulombic efficiency (CE), induced by the side reactions and the formation of a SEI film. Therefore, it is necessary to control the type and content of surface functional groups of B-d-CMs by selecting appropriate biomass precursors, pyrolysis conditions, and activation process.

**Figure 6:**
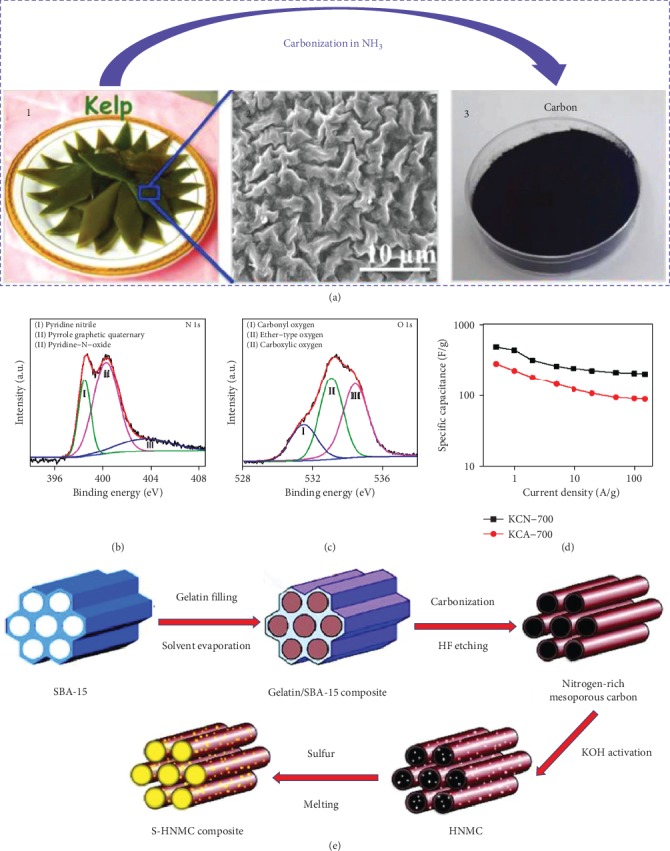
(a) Graphic illustration of pyrolysis process of kelp in the NH_3_. (b) N1s, (c) O1s XPS profiles, and (d) specific capacitances of kelp-derived carbon [118] (copyright 2015, American Chemical Society). (e) Graphic illustration of the preparation procedure of S/nitrogen-rich mesoporous carbon [119] (copyright 2014, Elsevier).

Ou et al. [124] developed ox horn that is composed of abundant C, N, and O elements as the precursor for preparing N- and O-enriched 3D carbon without any extra N source as the electrode material for SIBs. Attributed to the abundant nitrogen (5.5%) and oxygen (6.9%) functional groups which could introduce fast surface adsorption (pseudocapacitive behavior) on electrode materials, the as-obtained carbon materials showed a high initial reversible capacity and long cycling durability. Li and coauthors [118] reported a direct pyrolysis of kelp at 700°C in NH_3_ atmosphere, forming the O- (8.76 at%) and N- (5.04 at%) enriched B-d-CM with a three-dimensional structure as shown in Figures 6(a)–6(c). These abundant functional groups containing N and O on the surface of 3D carbon from kelp could introduce fast surface adsorption (pseudocapacitive process) on the surface of the electrode for EDLC (Figure 6(d)). Qu et al.'s group [119] prepared nitrogen-rich (9.74%) mesoporous carbons from gelatin through pyrolysis and subsequent KOH activation process for Li-S batteries (Figure 6(e)). The nitrogen functional groups could improve the efficient storage sites and diffusion kinetics by immobilizing sulfur and reduce the dissolution of polysulfide intermediates, delivering a high initial discharge capacity and long-term cycling stability.

**Figure 7:**
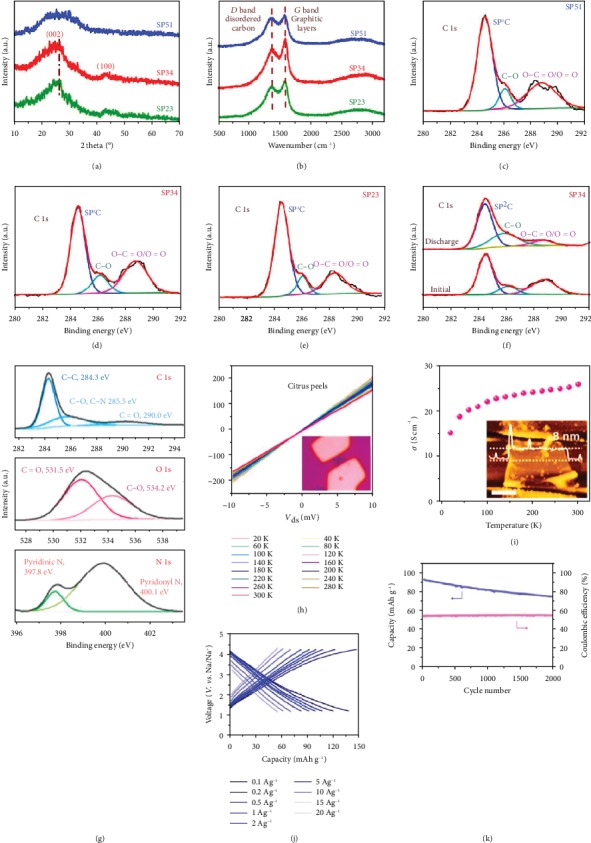
(a) XRD patterns, (b) Raman spectra, (c–e) C 1s XPS spectrum, and (f) C 1s XPS spectra after discharge of shaddock peel-derived hard carbon [125] (copyright 2019, Elsevier). (g) XPS spectra, (h) temperature-dependent current−voltage characteristics, (i) conductivity curve, (j) charge/discharge profiles, and (k) cyclic performance of the citrus peel-derived carbon [127] (copyright 2016, American Chemical Society).

Researchers are facing a dilemma between obtaining high content of surface functional groups and abundant pseudographitic structure, owing to the fact that low reaction temperature is favorable for maintaining more functional groups, while the enhanced graphitization degree of B-d-CMs usually requires high temperature. Therefore, it is very important to balance the content of surface functional groups and pseudographic structure in B-d-CMs when designing EES devices with enhanced storage sites and diffusion kinetics. For example, in SIBs, C=O groups could induce more active sites and reversible redox reaction via C = O + Na^+^ + e^−^⟷C − O − Na. The ordered pseudographitic structure could provide Na^+^ storage sites via intercalation and also accelerate the Na^+^ and electron transportation rate at the carbon electrode. As such, although some electrodes possessed a high amount of surface C=O groups, the unfavorable synthetic process for obtaining sufficient pseudographic structures enables it to display poor Na^+^ storage properties. Li et al.'s group has reported the work on controlling C=O and pseudographitic structure in hard carbon from the shaddock peel by a hydrothermal pretreatment and subsequent KOH-assisted pyrolysis procedure to enhance sodium storage properties (Figures 7(a)–7(f)) [125]. The obtained hard carbon materials can be controlled with different C=O contents of 51, 34, and 23% (labeled as SP51, SP34, and SP23, respectively). Although the C=O content of SP34 was lower than that of SP51, SP34 delivered a higher reversible capacity of 380 mAh g^−1^ at 50 mAg^−1^ after 500 cycles owing to the synergistic effect of C=O and more ordered pseudographic structure. Wang et al.'s work [126] also gave a constructive suggestion, in which sheet-like carbon particles interconnected into 3D micron-sized macropores were fabricated from willow catkins. The sample prepared at 700°C contained higher content of N (2.51 wt%) and O (13.28 wt%) groups than that of the sample derived from 800°C, while its degree of graphitization was higher than that prepared from 600°C, resulting in numerous storage sites and fast diffusion kinetics. When evaluated as an electrode for supercapacitors, it demonstrated a superior electrochemical performance.

Besides, surface functional groups can also improve the electronic conductivity. For example, nanoporous carbon nanosheets (NP-CNSs) [127] containing abundant active functional groups (C/O: 5.5, C/N: 34.3) were synthesized from citrus peels by Kim et al.'s group in Figures 7(g)–7(k). N groups could improve the electron transfer of NP-CNSs by n-type doping effects, exhibiting an electrical conductivity of 2.6 × 101 S cm^−1^, which is approximately 50 times higher than that of reduced graphene oxide. Gao and coworkers [85] used chitin as precursor via the hydrothermal method and then carbonized it into N-/O-enriched porous carbon nanosheets. The percentage of nitrogen and oxygen in carbon nanosheets was 8.12 at% and 6.69 at%. The nitrogen groups can enhance electronic conductivity (8.72 × 10^−2^ S cm^−1^), which favored electron transport during the charge/discharge process in SIBs.

### 2.4. Heteroatoms Doping Structure

Doping heteroatoms into carbon lattice can significantly improve the number of efficient storage sites and the level of diffusion kinetics. Firstly, electrochemical activity of the doped carbon either as nucleation/anchoring sites for electrolyte adsorption/desorption or as redox-reaction sites is highly improved, leading to large enhanced pseudocapacitance in EES devices [130]. Secondly, the interlayer spacing would be enlarged by heteroatom doping, resulting in increased specific capacity with more ion intercalation. Representative heteroatoms including N, S, B, P, and F could be incorporated in either a single- or codoped way to modify the B-d-CMs.

**Figure 8:**
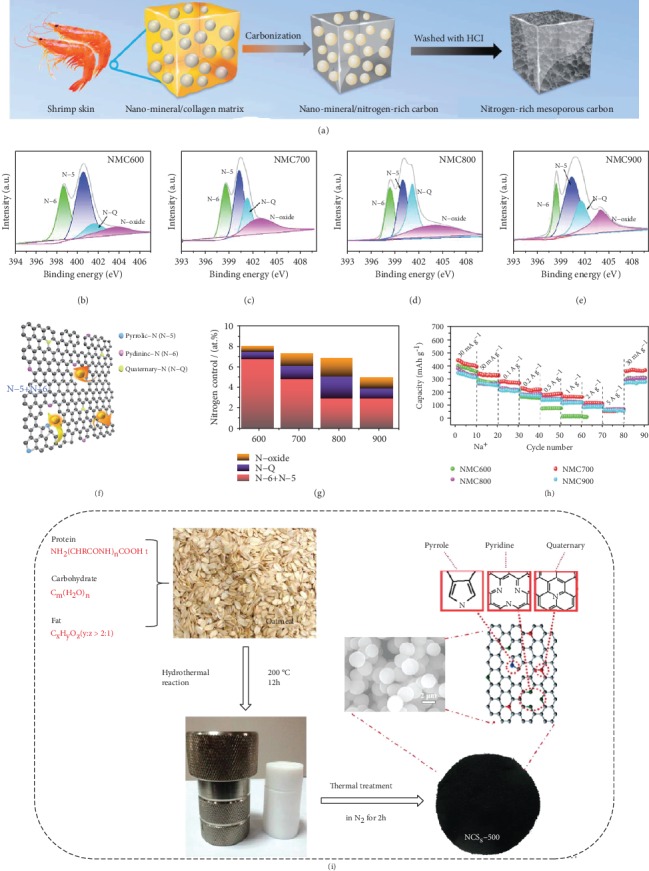
(a) Graphic illustration of the fabrication process, (b–e) N 1s spectra, (f) graphic structure of the binding conditions of N and the mechanism of Na^+^ storage, (g) the content of nitrogen species, and (h) rate performance of shrimp skin-derived N-rich carbons [128] (copyright 2017, Royal Society of Chemistry). (i) Graphic illustration of the preparation of oatmeal-derived N-doped carbon microspheres [129] (copyright 2016, Elsevier).

N is one of the most widely studied heteroatoms for B-d-CMs. Specifically, N could be introduced into the carbon frameworks in different configurations, including pyrrolic-nitrogen (N-5), pyridinic-nitrogen (N-6), and quaternary-nitrogen (N-Q) [3, 131]. Notably, the N configuration has significant influence on the activity and structure of B-d-CMs. N-5 exposes the planar edge or defect sites where they are located. N-6 is formed by replacing C with N at the defect sites or edges in the plane. The edge plane and defect sites can promote the ion diffusivity and electrical conductivity, while thus N-5 and N-6 can introduce highly chemical-active sites, enhancing surface adsorption capacity [132]. The N-Q can enhance electronic conductivity, which favors electron transport and ion diffusion during the charge/discharge process [85]. Typically, N could be incorporated into carbon frameworks by direct pyrolysis of N-containing carbon precursors. For example, N-rich mesoporous carbon materials (NMCs) [128] were obtained by annealing shrimp skin under a N_2_ atmosphere followed by hydrochloric acid washing, as shown in Figure 8(a). From the N 1s XPS spectra of the NMCs (Figures 8(b)–8(e)), N was incorporated into the B-d-CMs in four forms: the N-5, N-6, oxidized nitrogen (N-oxide), and N-Q. Quantified results (Figures 8(f) and 8(g)) showed that N-6 and N-5 were always the dominant configurations in prepared samples. When used as anodes for SIBs, the NMCs prepared at 700°C exhibited outstanding performance (Figure 8(h)). The higher performance compared with the other samples obtained at different pyrolysis temperatures was due to the combination of high content of N-6 and N-5 and high content of total nitrogen doping (7.26 at%), which was capable of enhancing the electronic conductivity of carbon, favoring adsorption process. Yan et al. [129] employed oatmeal as biomass precursors to prepare N-doped carbon microspheres (NCSs), as shown in Figure 8(i). Among the samples prepared at different pyrolysis temperatures, the NCSs prepared at 500°C had the highest content of nitrogen doping (4.1%) with 93.7% in the form of N-5 and N-6 and could afford more active sites and fast diffusion kinetics and hence enhance the capacity and electrical conductivity, delivering a larger capacity of 336 mAh g^−1^ after 50 cycles at 50 mA g^−1^ and excellent cycling stability and rate capacity.

In Li-S batteries, N dopant can also enhance the storage sites and diffusion kinetics by inhibiting the diffusion of soluble polysulfides and delaying the shuttle effect [133]. Zhang and coauthors [134] prepared N-doped carbon derived from silk fibroin protein encapsulating S as a high-performance cathode material for Li-S batteries. The high content of N doping was demonstrated to be very helpful in adsorbing sulfur species to effectively improve the electrochemical performance of sulfur cathodes. Geng et al.'s group [135] developed a two-step method via HNO_3_ and NH_3_ to prepare corncob-derived N-doped nanoporous carbon materials for Li-S batteries. It demonstrated that efficient N species played more crucial roles than total N content on the electrochemical performance of C/S composite cathodes. Specifically, N-6 and N-5 groups had positive roles in suppressing the diffusion of polysulfides and improving the adsorption ability of the carbon materials. Although the synthesized N-doped carbon at 600°C had smaller N content (4.12 wt%) than other samples at 400°C (4.19 wt%) and 800°C (6.11 wt%), it had a higher content of N-5 (60.74 at%) and N-6 (32.8 at%) than samples at 400°C (30.07, 23.53 at%) and 800°C (53.21, 30.85 at%), exhibiting the much improved cycling performance, which was about 3.5 times as that of nitrogen-free carbon.

**Figure 9:**
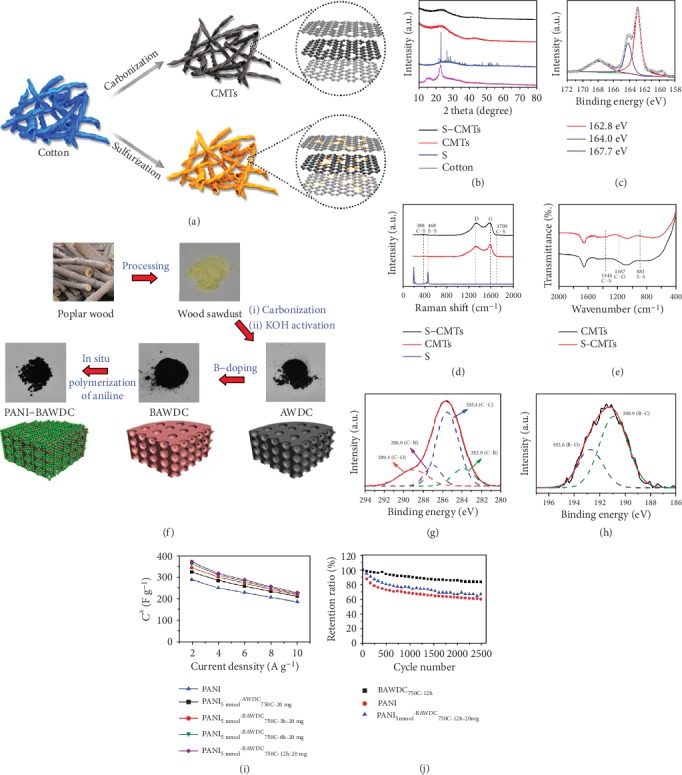
(a) Graphic representation of the synthetic procedure, (b) XRD patterns, (c) S 2p XPS spectrum, (d) Raman spectra, and (e) FTIR spectra of sulfur-doped carbon microtubes [136] (copyright 2018, American Chemical Society). (f) Synthesis process and structural schematic diagram, (g) C 1s XPS spectrum, (h) B 1s XPS spectrum, (i) different boron-doping time, and (j) cycling performances of B-doped activated wood-derived carbon [87] (copyright 2015, American Chemical Society).

S is an electroactive element with higher capacity and more reversible than O when reacting with ion, such as C − S + Na^+^ + e^−^⟷C − S − Na, offering extra storage sites for ions. Specifically, S would be doped into B-d-CMs in two forms: the overwhelming thiophene-type S and the oxidized-type S. The thiophene-type S could enlarge the interlayer distances due to the larger electrostatic repulsion force and elevate the conductivity of B-d-CMs, significantly improving the ion-diffusion and electron-transportation kinetics. It also offers more space to reduce the volume expansion and lower the adsorption energy [137]. As such, S doping is particularly promising in increasing specific capacity and rate performance of EES devices. For example, Wang et al. synthesized sulfur-doped carbon microtubes (S-CMTs) [136] with a S content of 10.2 wt% through a 700°C sulfurization of cotton roll for SIBs, as shown in Figure 9(a). XRD patterns (Figure 9(b)) indicated that S doping expanded the interlayer distances of the (002) planes of CMTs from 3.73 to 3.81 Å, which could boost the Na^+^ insertion/extraction process and improve the electrochemical activity. S 2p XPS, Raman, and FTIR spectra (Figures 9(c)–9(e)) confirmed that S was covalently bonded into the carbon framework of CMTs. The as-prepared S-CMTs showed a large charge capacity, outstanding rate capability, and exceptional cycling stability. Hao and coworkers [91] prepared ginkgo leaf-derived S-doped carbon materials (8.245 wt%) via a combined procedure of hydrothermal treatment in sulfuric acid solution and KOH activation. When applied in supercapacitors, it showed a high specific capacitance and only 2% of capacitance loss after 30000 cycles. In Li-S batteries, the C-S framework could also enhance the affinity between polysulfides and B-d-CMs, thus favorable for promoting immobilization of polysulfide ions so as to enhance the efficient storage sites and diffusion kinetics of Li-S batteries. Yang et al.'s group [90] synthesized a free-standing S-doped microporous carbon (SMPC) for Li-S batteries from luffa sponge. The in situ S doping microporous carbon (2.72 at%) demonstrated a high electrical conductivity of 1.89 S cm^−1^. Owing to its rich S doping, the as-prepared SMPC not only was conducive to the rapid transmission of electron and Li^+^ but also contributed to the enhancement of the affinity and binding energy of polar polysulfides with nonpolar carbon frameworks, delivering a high initial reversible capacity, superior rate capability, and excellent cycling stability.

Similar to N and S doping, B, P, and F doping also can enhance the number of efficient storage sites and the level of diffusion kinetics of B-d-CMs. B enters into carbon frameworks in the form of trigonal coordination, offering as an electron acceptor and modifying the electronic structure of B-d-CMs because its three valence electrons can induce a shift in the Fermi level to the conduction band [81]. The change of electronic structure of B-d-CMs would affect the storage sites and diffusion kinetics of EES devices. Notably, even a low level of B doping showed the catalytic influence on oxygen chemisorption process, resulting in enhanced redox reactions associated with O-containing functional groups on the surface of carbon [138]. As an example, B-doped activated wood-derived carbon-supported polyaniline particles were synthesized as electrode materials for high-performance supercapacitors, as shown in Figures 9(f)–9(j) [87]. P doping is also beneficial for stabilizing and modifying the structure of B-d-CMs. Firstly, P doping produces some P-functional groups on B-d-CMs, improving wettability of B-d-CMs which is conducive to penetration of electrolyte into the electrode materials [139]. Secondly, P=O is electrochemical redox active and can supply electroactive sites to improve its pseudocapacitance [140]. Thirdly, because the electronegativity of P is lower than that of carbon, C-P can change the charge and spin densities of carbon materials, together with the bigger size of P atom than carbon leading to structural defects in the carbon framework. This could further act as active sites in the electrochemical process [130]. For instance, Huang et al.'s group [141] developed P-rich carbons (13.3 at%) by H_3_PO_4_ activation of coffee grounds. The as-fabricated sample in supercapacitors showed a high energy density of 15 Wh kg^−1^ at a power density of 75 W kg^−1^. F is the element with the highest electronegativity, and F-doped carbon is capable of forming F-C bond with high polarity and stability, which would easily enrich the repulsive interaction between the carbon layers, thus expanding the interlayer spacing for more storage sites and excellent diffusion kinetics [142]. Wang et al. [95] synthesized F-doped carbon particles (1.1 at%) through direct pyrolysis and air activation of F-rich lotus petioles as anode materials for SIBs. The as-obtained electrode displayed an initial charge capacity of 230 mAh g^−1^ at a current density of 50 mA g^−1^. Although most B-d-CMs are rich in natural heteroatoms, which can significantly improve electrochemical performance, their content is low, and additional introduction process is still necessary. In addition, some B-d-CMs contain a few heteroatoms (e.g., Ca, Si, and Al) that affect performance and destroy the carbon structure, which needs to be removed.

**Figure 10:**
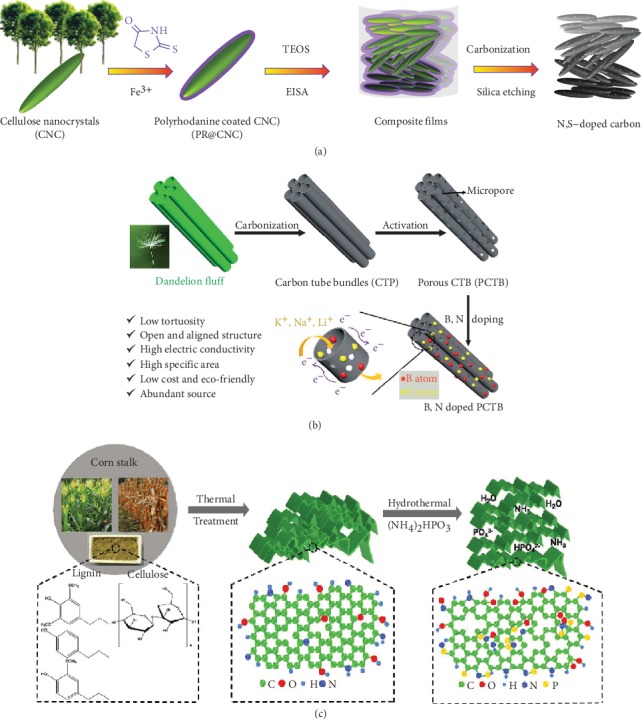
The schematic illustration of the fabrication of (a) N, S codoped cellulose [143](copyright 2015, Wiley), (b) N, B codoped carbon tube bundles [144] (copyright 2017, Royal Society of Chemistry), and (c) N, P codoped carbon sheets [145] (copyright 2018, Elsevier).

Compared with a single heteroatom dopant that merely improves one aspect of properties, codoping could obviously improve the efficient storage sites and diffusion kinetics of the B-d-CM electrodes to a larger extent based on the synergetic effects [68] of multiple heterogeneous elements. Since N doping could greatly elevate the electronic conductivity and diffusion kinetics of carbon, so, B, S, O, or P doping could enhance the pseudocapacitance, expand the interlayer distance, and enrich storage sites, N, O codoping [118, 122, 146, 147]; N, S codoping [33, 38, 143, 148, 149]; N, B codoping [31, 144, 150]; and N, P codoping [9, 145, 151–153] have been applied to further enhance the electrochemical performances of B-d-CMs. For example, Pangr and coworkers [143] synthesized the N, S-doped nanoporous carbon for advanced Li-S batteries via coating polydopamine and tetraethyl orthosilicate and pyrolysis as well as mixed with sulfur from biomaterial-derived cellulose nanocrystals, as presented in Figure 10(a). The codoping with S (3.2 at%) and N (2.4 at%) atoms in carbon frameworks significantly improved the chemisorption of lithium polysulfides and greatly enhanced the electric conductivity, favoring excellent reversible storage sites and high-rate kinetics. Zhao et al. [144] designed B and N codoped porous carbon materials by direct pyrolysis of dandelion fluff (Figure 10(b)). The incorporation of N (2.2 at%) and B (4.6 at%) heteroatoms into obtained carbon could induce double-layer capacitance and extra pseudocapacitance to improve the overall storage sites. Benefiting from the unique structure and N, B doping, the electrode when applied in supercapacitors exhibited a superior volumetric energy density of 12.15 Wh L^−1^ at 699.84 W L^−1^. Qin et al. [145] prepared N, P codoped carbon sheets from the rinds of corn stalks though a hydrothermal process using the (NH_4_)_2_HPO_4_ as N and P source (Figure 10(c)). It demonstrated that P (1.82 at%) and N (0.9 at%) atoms were well introduced into the carbon framework, which enlarged the interlayer distance and induced the improvement of electron transport and surface wetting ability of B-d-CMs with electrolytes. When applied to the Na^+^ storage, the as-obtained carbon displayed a stable discharge specific capacity. The comparison of surface functional groups and heteroatom doping of B-D-CMs and their electrochemical performance in various EES devices are presented in Table 2.

### 2.5. Composite Structure

Compositing B-d-CMs with high-capacity materials is also an effective way to enhance the storage sites [65]. Specifically, transition metal oxides are one group of the most promising candidates [154–157]. On the one hand, based on the alloy, insertion, and conversion reactions, transition metal oxides possess high capacities (e.g., above 1000 mAh g^−1^ in SIBs) and could largely increase the storage sites for composite electrode materials [65, 68]. On the other hand, owing to the high specific surface area, hierarchically porous structure and rich heteroatoms, the B-d-CMs have abundant pathways for ion diffusion and electron transportation, improving the diffusion kinetics of composite electrode materials. Yang and Park [158] prepared MnO_2_/banana peel-derived porous carbon (BPC) composites for supercapacitors. The charge storage process of MnO_2_ included H^+^ intercalation/deintercalation reaction and H^+^ surface adsorption/desorption in redox reaction, which greatly increased the storage sites of composite materials. The 3D BPC substrate with the hierarchically porous structure provided the growth space for MnO_2_, as well as promoted the ion diffusion of electrolyte and served as a conductive network for electrons, showing a good cycling stability with a capacitance retention ratio of 92.3% after 1000 cycles (at 1 A g^−1^). Shi et al. [159] synthesized a hierarchical nanostructure of Co_3_O_4_@biomass-derived carbon fiber@Co_3_O_4_ for high-performance supercapacitors. The Co_3_O_4_ particles were well coated onto both the inner and outer surface of the porous fiber wall. The carbon framework could overcome the kinetic limitations of both ions and electrons, while Co_3_O_4_ particles exposed on both surfaces maximized Faradaic processes and redox reactions with uniformly dispersed active materials.

**Figure 11:**
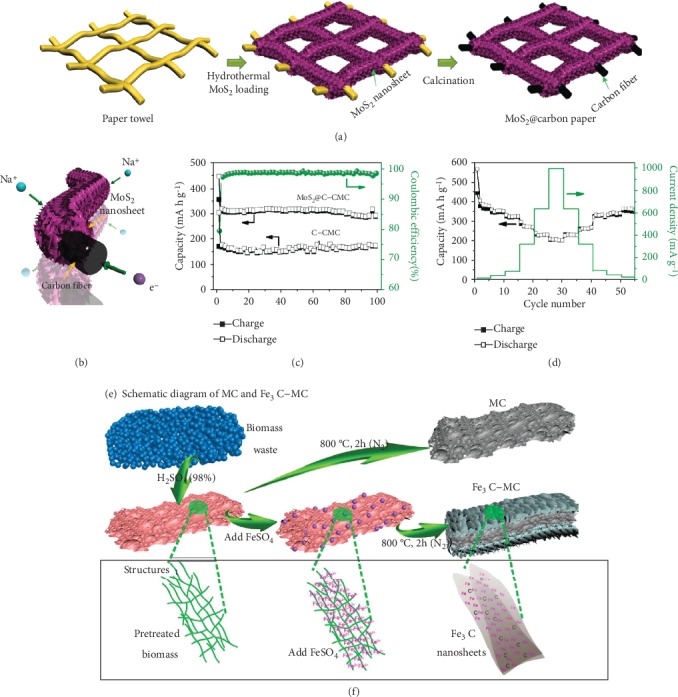
Graphic illustration (a) for the fabrication, (b) showing pathways for Na^+^ diffusion and electron transfer, (c) cycling, and (d) rate performance of the MoS2 vertically aligned on carbon paper. (e) The schematic diagram for synthesis of mesoporous carbon (MC) and Fe_3_C-MC [161] (copyright 2016, Wiley). (f) Structural schematic diagram for the formation of Fe_3_C nanosheets [167] (copyright 2019, Elsevier).

Besides metal oxides, incorporation of metal sulfides into B-d-CMs to form a composite electrode has also gained much attention in EES devices, due to their enlarged layered structure, redox variabilities, and high structure stabilities, which could provide more storage sites [160]. For instance, Xie and coworkers [161] designed and fabricated MoS_2_ nanosheets vertically aligned on paper towel-derived carbon paper as a freestanding electrode for high-performance reversible SIBs (Figures 11(a)–11(d)). MoS_2_ nanosheets vertically aligned on the carbon paper substrate interwoven randomly from 1D carbon fiber, constructing interconnected ionic and continuous electronic transfer pathways without volume expansion during charge/discharge process. The as-fabricated freestanding electrodes showed a high reversible capacity, high initial CE, high rate performance, and long cycling life. Li et al.'s group [162] developed 1D porous FeS/carbon fiber micro-/nanostructures as high-capacity and durable anodes for SIBs via pyrolysis of double-helix-structured Fe-carrageenan fibers. The FeS nanoparticles could provide more Na^+^ storage sites, significantly enhancing the Na^+^ storage performance. The 1D porous carbon fibrous matrix could effectively improve the structural stability during charge/discharge process and improve Na^+^ and electron transport kinetics, guaranteeing an outstanding rate and cycling performance.

In addition to transition metal oxides and sulfides, B-d-CMs have also been composited with other components to create novel composite electrodes with more storage sites and faster diffusion kinetics [163–168]. For example, Li et al.'s group [167] reported an ultrathin (~1 nm) Fe_3_C nanosheets growing on mesoporous carbon (Fe_3_C-MC), as shown in Figures 11(e) and 11(f). Biomass waste corncob was used as the carbon source, which was pretreated by H_2_SO_4_ and then mixed with ferrous sulfate followed by annealing at 800°C. In the Li-S batteries, the Fe_3_C nanosheets of Fe_3_C-MC composites played a significant role in the adsorption and conversion of polysulfides, and electronic transmission. Yu and coworkers [168] synthesized a graphene-wrapped hair-derived carbon/S composite for high-performance Li-S batteries, showing a high initial discharge capacity of 1113.2 mAh g^−1^ at 0.2 C. Such high performance was mainly associated with the fact that introducing graphene effectively speeded up the electron transportation and Li^+^ diffusion, along with the hard carbon derived from hair with nitrogen doping, further restrained the shuttle effect of lithium polysulfides. The comparison of composite structures of B-d-CMs and their electrochemical performance in various EES devices are displayed in Table 3.

## 3. Conclusions and Outlook

This review reported B-d-CMs as a kind of sustainable and green electrodes in EES devices and summarized various mechanisms for enhancing the number of efficient storage sites and the level of diffusion kinetics from the aspect of structural control ranging from pseudographic structure and hierarchical pore structure to surface functional groups, heteroatom doping, and composition of B-d-CMs with other electrode materials, thus improving their electrochemical performance in SIBs, Li-S batteries, and supercapacitors.

Specifically, the pseudographitic structure in B-d-CMs can not only provide the enlarged interlayer spacing for ion insertion/extraction but also accelerate the ion diffusion and electron transportation via high crystallinity. The hierarchical pore structure has different functions in various EES devices. In SIBs, it could supply superior access of the electrolyte to the B-d-CM surface shortening the Na^+^ diffusion pathway and increase the specific surface area improving the efficient absorbing sites. For supercapacitors, it could increase the pseudocapacitors by offering fast ion/electron transfer kinetics and alleviating structural degradation during the charge/discharge process. In Li-S batteries, it could form an electron-conducting network to trap the soluble polysulfide intermediates, which effectively elevates the utilization efficiency of sulfur for Li^+^ reaction, as well as enhances the diffusion kinetics of Li^+^ by reducing the viscosity of electrolyte. The surface functional groups of B-d-CMs could provide numerous electrochemical storage sites for efficient ion adsorption/desorption and participate in surface redox reaction facilitating more storage sites, faster ion diffusion, and smaller electrode structure being destroyed than intercalation reaction. Doping heteroatoms into the carbon lattice of B-d-CMs could introduce extra storage sites (electroactive sites and defects), favoring electron transport and ion diffusion and increasing the repulsive interaction between carbon layers, which would improve the adsorption/desorption process, promote electronic conductivity and diffusion kinetics, and expand the interlayer distance. The composite structure of B-d-CMs and other electrode materials could enhance storage sites and diffusion kinetics through the synergic effect: due to the rich heteroatoms, hierarchical pores, and high specific surface area, the B-d-CMs have abundant pathways for ion diffusion and electron transfer, improving the diffusion kinetics of composite electrodes; based on the alloy, insertion, and conversion reaction, the metal compound possesses high capacities above 1000 mAh g^−1^, which could largely increase the storage sites for composite electrode materials.

From Tables 1–3, it could be concluded that controlling the hierarchically porous structure and composite structure has a more obvious effect on improving storage sites and diffusion kinetics, compared with other structure control strategies. The reason may be that the hierarchical pore structure can significantly increase the adsorption capacity by increasing the specific surface area and diffusion kinetics. And the composite structure can significantly increase the capacity through the introduction of high-capacity materials. Although some progress about enhanced storage sites and diffusion kinetics in B-d-CMs for the field of EES devices have been made, there are still some challenges:

1. The structure of B-d-CMs is determined by biomass raw materials. The biomass precursor needs to be further explored, such as using waste, easy accessibility, and specially structured biomass, instead of costly and rare biomass
2. The synthesis condition enabling highly ordered pseudographitic structures will result in a small specific surface area, a narrow interlayer spacing, and a few amorphous carbon region and surface functional groups, exhibiting less storage sites and poor diffusion kinetics. Therefore, it is necessary to develop strategies to obtain pseudographite structure under the condition favorable for large specific surface area, interlayer spacing, amorphous region, and sufficient surface functional groups
3. Hierarchically porous structure needs to be further designed. Firstly, 3D-interconnected hierarchically porous structured B-d-CMs should be paid more attention, because 3D structure is very beneficial for shortening the diffusion distance and improving diffusion kinetics during charge/discharge process especially at high rate. Secondly, the hierarchically porous structure is generally produced from the activation process, leading to a high BET area. However, the high surface area makes many irreversible reactions easy to generate, resulting in a low initial CE. Thus, this requires increasing the specific surface area without destroying the initial CE
4. Compared with single heteroatom doping that elevates only one aspect of properties, co-/polyatomic doping can improve the integral performance of the B-d-CMs because of their synergic effects. However, most of the studies are focused on N-doped and its co-doped B-d-CMs, which should be extended to other atoms codoping
5. Currently, B-d-CMs as support skeletons could load high-capacity materials with more storage sites, while how to address the issue of interface combination is significantly essential
6. It is necessary to explore synthetic technologies for large-scale production of B-d-CM electrodes to expand their application into the actual industrial applications

## Figures and Tables

**Table 1:** Comparison of the pseudographitic structure and hierarchical pore structures of B-d-CMs and their electrochemical performance in various EES devices.

Biomass materials	*d*_002_ (nm)	*I_G_*/*I_D_*	*S*_BET_ (m^2^ g^−1^)	*V*_t_ (cm^3^ g^−1^)	Hierarchical porous structure	Energy applications	Capacity (current density)	Capacity (high current density)	Ref.
Banana peel	0.39	0.92	130	0.19	No	SIBs	355 mAh g^−1^ (50 mA g^−1^)	238 mAh g^−1^ (0.5 a g^−1^)	[28]
Shaddock peel	0.38	0.57	538	—	No	SIBs	287.3 mAh g^−1^ (50 mA g^−1^)	205.6 mAh g^−1^ (0.5 a g^−1^)	[104]
Pith/chitosan	0.57	1.00	1786	0.82	No	Supercapacitors	339 F g^−1^ (0.25 a g^−1^)	280 F g^−1^ (100 a g^−1^)	[101]
Cornstalk	0.34	1.40	788	0.63	No	Supercapacitors	—	213 F g^−1^ (1 A g^−1^)	[102]
Peanut skin	0.37	—	2500	1.69	Yes	SIBs	431 mAh g^−1^ (0.1 a g^−1^)	47 mAh g^−1^ (10 a g^−1^)	[29]
Prawn shell	—	—	336	—	Yes	SIBs	325 mAh g^−1^ (0.1 a g^−1^)	107 mAh g^−1^ (2 a g^−1^)	[44]
Natural silk	—	1.15	2494	2.28	Yes	Supercapacitors	242 F g^−1^ (0.1 A g^−1^)	155 F g^−1^ (10 A g^−1^)	[113]
Cotton stalk	—	—	1481	1.21	Yes	Supercapacitors	114 F g^−1^ (0.5 a g^−1^)	98 F g^−1^ (2 a g^−1^)	[114]
Rice husks	—	—	525	0.49	Yes	Li-S batteries	1023 mAh g^−1^ (1.0 C)	500 mAh g^−1^ (5.0 C)	[80]
Soybean	—	—	1500	0.70	Yes	Li-S batteries	950 mAh g^−1^ (0.2 C)	460 mAh g^−1^ (0.5 C)	[115]

**Table 2:** Comparison of surface functional groups and heteroatom doping of B-d-CMs and their electrochemical performance in various EES devices.

Biomass materials	Functional groups/heteroatom doping	Energy applications	Capacity (low current density)	Capacity (high current density)	Ref.
Cherry petals	O (12.0 at%) N (1.4 at%)	SIBs	310.2 mAh g^−1^ (20 mA g^−1^)	146.5 mAh g^−1^ (0.5 a g^−1^)	[118]
Longan shell	O (6.27 wt%) N (1.36 wt%)	SIBs	345.9 mAh g^−1^ (0.1 a g^−1^)	304.2 mAh g^−1^ (5 A g^−1^)	[119]
Ox horn	O (6.90 at%) N (5.50 at%)	SIBs	419 mAh g^−1^ (0.1 a g^−1^)	117 mAh g^−1^ (5 a g^−1^)	[122]
Enteromorpha	O (11.36 at%) N (0.74 at%)	Supercapacitors	201 F g^−1^ (1 a g^−1^)	122.6 F g^−1^ (100 A g^−1^)	[120]
Kelp	O (8.76 at%) N (5.04 at%)	Supercapacitors	440 F g^−1^ (0.5 a g^−1^)	180 F g^−1^ (150 a g^−1^)	[123]
Willow catkins	O (13.28 wt%) N (2.51 wt%)	Supercapacitors	340 F g-^1^ (0.1 a g^−1^)	231 F g^−1^ (10 a g^−1^)	[126]
Gelatin	N (9.74 at%)	Li-S batteries	1209 mAh g^−1^ (1 C)	595 mAh g^−1^ (3 C)	[124]
Shrimp skin	N (7.26 at%)	SIBs	434.6 mAh g^−1^ (30 mA g^−1^)	110 mAh g^−1^ (2 a g^−1^)	[131]
Pomelo Peel	N (3.90 at%)	Supercapacitors	260 F g^−1^ (1 a g^−1^)	44 F g^−1^ (10 a g^−1^)	[129]
Cotton	S (10.2 wt%)	SIBs	532 mAh g^−1^ (200 mA g^−1^)	234 mAh g^−1^ (2 a g^−1^)	[137]
Ginkgo leaves	S (8.25 wt%)	Supercapacitors	364 F g^−1^ (0.5 a g^−1^)	245 F g^−1^ (40 a g^−1^)	[91]
Luffa sponge	S (2.72 at%)	Li-S batteries	1544 mAh g^−1^ (0.2 C)	781.2 mAh g^−1^ (5 C)	[90]
Poplar wood	B (3.70 at%)	Supercapacitors	372 F g^−1^ (2 a g^−1^)	251 F g^−1^ (10 a g^−1^)	[87]
Coffee bean	P (13.3 at%)	Supercapacitors	180 F g^−1^ (0.05 a g^−1^)	157 F g^−1^ (1 a g^−1^)	[141]
Lotus petioles	F (1.1 at%)	SIBs	230 mAh g^−1^ (50 mA g^−1^)	228 mAh g^−1^ (200 mA g^−1^)	[95]
Cellulose	N (2.4 at%) S (3.2 at%)	Li-S batteries	1370 mAh g^−1^ (C/20)	830 mAh g^−1^ (2 C)	[145]
Dandelion fluff	N (2.2 at%) B (4.6 at%)	Supercapacitors	355 F g^−1^ (1 a g^−1^)	292 F g^−1^ (20 a g^−1^)	[149]
Corn stalks	N (0.90 at%) P (1.82 at%)	SIBs	233 mAh g^−1^ (0.1 a g^−1^)	143 mAh g^−1^ (1 A g^−1^)	[153]

**Table 3:** Comparison of composite structures of B-d-CMs and their electrochemical performance in various EES devices.

Sample	Biomass materials	Structure	Energy applications	Capacity (low current density)	Capacity (high current density)	Ref.
MnO_2_/C	Natural flax fiber	Fibrous	Supercapacitors	683.73 F g^−1^ (2 a g^−1^)	269.04 F g^−1^ (300 a g^−1^)	[154]
Fe_3_O_4_/C	Watermelon	3D porous	Supercapacitors	333.1 F g^−1^ (1 A g^−1^)	—	[155]
Co_3_O_4_/C	Dextran	Nanopetal like	Supercapacitors	400 F g^−1^ (0.5 A g^−1^)	175 F g^−1^ (2 a g^−1^)	[156]
MnO_2_/C	Hemp stem	3D hierarchical porous	Supercapacitors	340 F g^−1^ (1 a g^−1^)	300 F g^−1^ (30 a g^−1^)	[157]
MnO_2_/C	Banana peel	Hierarchical porous	Supercapacitors	139.6 F g^−1^ (300 mA g^−1^)	70 F g^−1^ (10 a g^−1^)	[158]
Co_3_O_4_/C/Co_3_O_4_	Raw cotton	Sandwich like	Supercapacitors	892 F g^−1^ (0.5 a g^−1^)	102 F g^−1^ (2.0 a g^−1^)	[159]
MoS_2_/C	Paper towel	3D hierarchical porous	SIBs	446 mAh g^−1^ (20 mA g^−1^)	102 mAh g^−1^ (10 a g^−1^)	[161]
Ni-doped CoS_2_/N,P-doped C	Yeast cells	Spherical	SIBs	407 mAh g^−1^ (100 mA g^−1^)	243 mAh g^−1^ (1000 mA g^−1^)	[164]
FeS/C	Fe-carrageenan fibers	Fibrous	Li-S batteries	283 mAh g^−1^ (1 A g^−1^)	247 mAh g^−1^ (5 a g^−1^)	[162]
Fe_3_C/C	Corncobs	Nanosheet	Li-S batteries	1530 mAh g^−1^ (0.1 C)	699 mAh g^−1^ (0.5 C)	[167]
